# The Flexible Nature of the Interaction Between Attention and Working Memory

**DOI:** 10.5334/joc.68

**Published:** 2019-08-08

**Authors:** Stefan Van der Stigchel, Christian N. L. Olivers

**Affiliations:** 1 Helmholtz Institute, Department of Experimental Psychology, Utrecht University, Utrecht, NL; 2 Department of Experimental and Applied Psychology, Institute for Brain and Behavior Amsterdam, Vrije Universiteit Amsterdam, Amsterdam, NL

**Keywords:** Working memory, Attention, Action

## Abstract

In the research literature, attention and working memory are often intimately linked. In his excellent target article, Oberauer concludes that attention is not a necessary requirement for working memory maintenance. In our reply, we argue that attention is an emergent property of maintaining the current task goal. Humans can flexibly transfer the storage of information between different states and different memory systems as the need arises. Only information in service of currently active task goals requires attentional resources for maintenance. In this state, memoranda can bias behavior and are susceptible to interference from additional attention demanding tasks.

When colleagues complain about their low attention span, they in fact complain about the difficulties in successfully maintaining a task goal in working memory for a prolonged period of time. Situations like these make it clear that not only in the research literature, but also in everyday conversation attention and working memory are often intimately linked. In his excellent target article, Oberauer (2019) evaluates the evidence and theoretical implications of the working memory – attention relationship, and paints a precise picture of the different strengths and gaps in our current knowledge. Citing results from, among others, studies showing that the dual-task costs evoked by additional attention-demanding tasks diminish over the course of the memory retention interval (e.g., Thalmann, Souza, & Oberauer, 2019) or are sometimes even not observed at all (e.g., Klapp, Marshburn, & Lester, 1983), Oberauer arrives at the conclusion that attention is not a necessary requirement for working memory maintenance and thus the two processes cannot be equated.

Although we agree with the view that attention and working memory can be dissociated, it leaves the question as to why many other studies suggest a clear interaction between working memory maintenance and attention. Specifically within the visual working memory literature, there is ample evidence for what has been dubbed the sensory recruitment hypothesis, which claims that temporary storage of visual information in visual working memory recruits the same sensory processing areas responsible for perception of physically present information. Besides efficient in terms of neural architecture, such an approach is also efficient in biasing visual attention and eye movements towards visual input matching memoranda (Olivers, Meijer, & Theeuwes, 2006; Silvis & Van der Stigchel, 2014) or to boost memory-matching information into visual awareness (Gayet, Paffen, & Van der Stigchel, 2013) – in fact, so efficient that VWM often drives attention automatically.

In our view, one fruitful perspective is to see attention as an emergent property of maintaining the current task goal (cf., Krauzlis, Bollimunta, Arcizet, & Wang, 2014). Under this view, activating a particular task goal will also activate the accompanying sensory representations required for accomplishing this goal, which behaviourally emerges as attention, and neurophysiologically emerges as the active firing of sensory representations known as sensory recruitment. At the action side, task goal maintenance will come with a readiness to respond, whether manually or through eye movements.

The other side of the coin is then that observers may also store memoranda not in service of a current task goal, or for which sensory recruitment is not beneficial to that goal. For instance, when visual interference is expected during the retention interval, visual working memory representations stored in the sensory processing areas could potentially be disrupted. In this situation, memoranda should be shielded from interacting with perception, and may be strategically recoded into non-visual memorization (Gayet, Paffen, & Van der Stigchel, 2018), such as verbal labels that might be less susceptible to visual interference (Olivers et al., 2006) or dual-task interference (Hazeltine, Ruthruff, & Remington, 2006).

In addition to recoding memoranda, humans may flexibly transfer memoranda from working memory system to long term memory (Reinhart & Woodman, 2013). For instance, memory-induced sensory biases diminish across repeated memorizations of the same item within just one or two trials, despite the fact that the memory itself clearly improves with learning (van Moorselaar, Theeuwes, & Olivers, 2016) (Gayet, van Moorselaar, Olivers, Paffen, & Van der Stigchel, 2019). Importantly, this shows that memory systems that do not involve sensory recruitment can rapidly take over. The same studies also found evidence that the system returns to active sensory recruitment when a new item is anticipated. Such long term storage mechanisms may be the same as those underlying what has become known as ‘passive’ or ‘silent’ working memory, when memoranda are stored not for the current but for prospective goals, presumably through changes in connectivity patterns (Lewis-Peacock, Drysdale, Oberauer, & Postle, 2012; van Loon, Olmos-Solis, Fahrenfort, & Olivers, 2018).

Given the efficiency of these strategies within visual working memory, it is perhaps no stretch to propose that the flexibility observed for visual information can also be applied to other types of to-be-remembered information, such as verbal or spatial information. The idea that the storage of information can flexibly transfer between different states and different memory systems as the need arises could also explain the lack of profound dual-tasks effects when performing additional attention-demanding tasks. This does not mean that there is no form of maintenance where attention is involved. We propose that only information in service of currently active task goals requires attentional resources for maintenance. Only in this state, memoranda can bias behavior, but in turn are susceptible to interference from additional attention demanding tasks.

## References

[1] Gayet, S., Paffen, C. L. E., & Van der Stigchel, S. (2013). Information matching the content of visual working memory is prioritized for conscious access. Psychological Science, 24(12), 2472–2480. DOI: 10.1177/0956797613495882 24121415

[2] Gayet, S., Paffen, C. L. E., & Van der Stigchel, S. (2018). Visual working memory storage recruits sensory processing areas. Trends in Cognitive Sciences, 22(3), 189–190. DOI: 10.1016/j.tics.2017.09.011 29050827

[3] Gayet, S., van Moorselaar, D., Olivers, C. N. L., Paffen, C. L. E., & Van der Stigchel, S. (2019). Prospectively reinstated memory drives conscious access of matching visual input. Scientific Reports, 9, 4793 DOI: 10.1038/s41598-019-41350-7 30886323 PMC6423023

[4] Hazeltine, E., Ruthruff, E., & Remington, R. W. (2006). The role of input and output modality pairings in dual-task performance: Evidence for content-dependent central interference. Cognitive Psychology, 52(4), 291–345. DOI: 10.1016/j.cogpsych.2005.11.001 16581054

[5] Klapp, S. T., Marshburn, E. A., & Lester, P. T. (1983). Short-term memory does not involve the “working memory” of information processing: The demise of a common assumption. Journal of Experimental Psychology: General, 112(2), 240–264. DOI: 10.1037/0096-3445.112.2.240

[6] Krauzlis, R. J., Bollimunta, A., Arcizet, F., & Wang, L. (2014). Attention as an effect not a cause. Trends in Cognitive Sciences, 18(9), 457–464. DOI: 10.1016/j.tics.2014.05.008 24953964 PMC4186707

[7] Lewis-Peacock, J. A., Drysdale, A. T., Oberauer, K., & Postle, B. R. (2012). Neural evidence for a distinction between short-term memory and the focus of attention. Journal of Cognitive Neuroscience, 24(1), 61–79. DOI: 10.1162/jocn_a_00140 21955164 PMC3222712

[8] Oberauer, K. (2019). Working Memory and Attention – A Conceptual Analysis and Review. Journal of Cognition, 2(1): 36, pp. 1–23. DOI: 10.5334/joc.58. PMC6688548 31517246

[9] Olivers, C. N. L., Meijer, F., & Theeuwes, J. (2006). Feature-based memory-driven attentional capture: visual working memory content affects visual attention. Journal of Experimental Psychology: Human Perception and Performance, 32, 1243–1265. DOI: 10.1037/0096-1523.32.5.1243 17002535

[10] Reinhart, R. M., & Woodman, G. F. (2013). High stakes trigger the use of multiple memories to enhance the control of attention. Cerebral Cortex, 24(8), 2022–2035. DOI: 10.1093/cercor/bht057 23448876 PMC4089381

[11] Silvis, J. D., & Van der Stigchel, S. (2014). How memory mechanisms are a key component in the guidance of our eye movements: evidence from the global effect. Psychonomic Bulletin & Review, 21(2), 357–362. DOI: 10.3758/s13423-013-0498-9 24002964

[12] Thalmann, M., Souza, A. S., & Oberauer, K. (2019). Revisiting the attentional demands of rehearsal in working-memory tasks. Journal of Memory and Language, 105, 1–18. DOI: 10.1016/j.jml.2018.10.005

[13] van Loon, A. M., Olmos-Solis, K., Fahrenfort, J. J., & Olivers, C. N. (2018). Current and future goals are represented in opposite patterns in object-selective cortex. ELife, 7, e38677 DOI: 10.7554/eLife.38677 30394873 PMC6279347

[14] van Moorselaar, D., Theeuwes, J., & Olivers, C. N. (2016). Learning changes the attentional status of prospective memories. Psychonomic Bulletin & Review, 23(5), 1483–1490. DOI: 10.3758/s13423-016-1008-7 26892228 PMC5050236

